# Comparative Clinical Presentation of Bronchial Asthma in Adults and Children in North India

**DOI:** 10.7759/cureus.108525

**Published:** 2026-05-08

**Authors:** Shreya Tripathi, Surya Kant, Ajay Verma, Jyoti Bajpai, Sarika Gupta, Prabhanshu Tripathi, Arpita Singh

**Affiliations:** 1 Department of Respiratory Medicine, King George's Medical University, Lucknow, IND; 2 Department of Respiratory Medicine, Dr. Ram Manohar Lohia Institute of Medical Sciences, Lucknow, IND; 3 Department of Pediatrics, King George's Medical University, Lucknow, IND; 4 FEST Division (Food, Drug &amp; Chemical, Environment and Systems Toxicology Division), Council of Scientific and Industrial Research (CSIR)-Indian Institute of Toxicology Research, Lucknow, IND; 5 Department of Pharmacology, Dr. Ram Manohar Lohia Institute of Medical Sciences, Lucknow, IND

**Keywords:** adult asthma, allergic rhinitis, asthma, asthma control, pediatric asthma

## Abstract

Background

Asthma is a chronic inflammatory airway disease with heterogeneous clinical expression influenced by age of onset, atopy, and environmental exposures. Age-related variations in symptoms, exacerbation history, asthma control, and lung function may affect treatment.

Objective

The primary objective of this study was to compare the clinical, allergic, and spirometric characteristics between pediatric and adult patients with asthma. The secondary objective was to evaluate asthma control status and related factors in both groups.

Methods

In our cross-sectional study, we enrolled 96 children (6-12 years) and 136 adolescents and adults (13-65 years) diagnosed with bronchial asthma via consecutive sampling in a tertiary care setting. Their diagnosis was based on the Global Initiative for Asthma (GINA) criteria and spirometric reversibility. Demographic details, comorbidities, trigger exposures, family history and atopy history, and asthma control (GINA Asthma Symptom Control Test) were recorded. Clinically stable patients with no current respiratory infection were included, while those with major chronic respiratory conditions, cardiac illness, or malignancy were excluded. Spirometry parameters were also compared between adult and pediatric patients. Spirometry was conducted by a trained technician (equipment: COSMED Spirometer, Rome, Italy) with proper calibration in accordance with the American Thoracic Society (ATS) guidelines.

Results

Allergic rhinitis was the most frequent comorbidity and was more common in asthmatic children than adults (62 (64.6%) vs 56 (41.2%)). Children had a higher prevalence of family history of asthma than in adults (43 (44.8%) vs 22 (16.2%)). Adults had greater exposure to smoking and biomass fuels and a higher proportion of late-onset asthma. Well-controlled asthma was observed in 26 (27.1%) children and 42 (30.9%) adults, while partly controlled asthma was almost similar in adults and in children (70 (51.5%) vs 43 (44.8%)). Adults demonstrated significantly lower predicted forced expiratory volume in one second (FEV₁%) and FEV₁/forced vital capacity (FVC) ratio than children (p < 0.0001), indicating more severe baseline airflow limitation.

Conclusion

Asthma shows distinct age-related differences in allergic comorbidity burden, exposure profiles, and lung function impairment. These findings support the need for tailored, age-specific management strategies to improve asthma control across the life course perspective.

## Introduction

Asthma is a long-term chronic inflammatory disease of the airways with many variations, representing different manifestation from one individual to the other. It is mainly characterized by episodic reversible airway obstruction that presents with a variable combination of intermittent symptoms like cough, wheezing (whistling sound while breathing), shortness of breath, or chest tightness [1]. It affects individuals in all age groups, imposing a social and economic burden on the individual as well as society. Asthma is more likely to occur if any other family members also have asthma, particularly a parent or sibling. It is a result of complex gene and environment interactions. Both genetic factors (genetic polymorphisms and family history) and environmental factors (tobacco smoke, air pollution, allergens) contribute to the development of asthma [2,3]. 

Prevalence of asthma

Asthma is a serious global health concern affecting about 300 million people around the world. This condition disturbs the quality of life and productivity of work in most of the patients [4]. Asthma prevalence has shown an increasing trend in high-income nations like North America and Central Europe over the past decades, whereas low- and middle-income countries experience higher death rates and greater burden of disability-adjusted life years (DALYs) [5]. In low-income or lower-middle-income countries like sub-Saharan Africa and South Asia, scarcity of healthcare facilities, economic challenges, and low health awareness lead to many preventable asthma deaths, whereas in high-income countries, better healthcare infrastructure aids in better asthma management [5,6]. A study in 2021 also reports that the USA, India, and China are the top three countries globally in asthma incident cases [6]. In India, a population-based survey reports a pooled prevalence of asthma to be 7.9 among children [7]. The recent Global Burden of Disease (GBD, 1990-2019) estimated the total burden of asthma in India as 34.3 million, accounting for 13.09% of the global burden [8].

Diagnosis of asthma is based mainly on history, physical examination, and lung function test. The Global Initiative for Asthma (GINA) guidelines emphasize the regular evaluation of daytime and nocturnal symptoms, reliever use, and activity limitation to categorize asthma as well-controlled, partly controlled, or uncontrolled and to guide stepwise pharmacologic and non-pharmacologic interventions. [9].

Age at onset of disease is a major factor in determining asthma phenotype. Early- or childhood-onset asthma is typically linked to atopy and allergic sensitization, with variable degrees of severity, whereas later- or adult-onset asthma shows a weaker association with allergy [10]. Furthermore, comorbid conditions and potential confounders like smoking, hormonal factors, infections, and occupational exposures may further alter the underlying immunoinflammatory mechanisms [11]. However, most available studies have evaluated either pediatric or adult populations separately, rather than studying a lifespan perspective that compares these groups simultaneously [12].

Asthma with allergy is common and may present at any age, but most often it starts in childhood, and these patients mostly have a family history of eczema, allergic rhinitis, or food or drug allergy [11]. Usually, exposure to allergens causes the initial symptoms, which involve increased inflammation of the airways. These patients present with eosinophilic airway inflammation and respond well to inhaled corticosteroids (ICS) [12]. Patients with non-allergic asthma typically respond to ICS in the short term. They may have a paucigranulocytic, eosinophilic, or neutrophilic sputum cellular profile. Moreover, non-allergic asthma is not associated with allergic sensitization and presents as a late-onset disease, with symptoms mostly beginning after the age of 40 years. It may become persistent, which makes it difficult to manage due to reduced responsiveness to corticosteroid treatment [12,13].

Due to fast urbanization, seasonal variation in aeroallergens, and air pollution, asthma triggers and clinical patterns may differ among age groups in North India. Therefore, our study aims to analyze both age groups (adults and children) simultaneously within the same geographical area. The primary objective of this study was to compare the clinical, allergic, and spirometric characteristics of pediatric and adult patients with bronchial asthma in a tertiary care setting in North India. The secondary objective was to evaluate and compare asthma control status and associated factors between these two groups. Through this study, we seek to provide valuable insights into age-related variation in asthma and its implications for future targeted management strategies [14].

## Materials and methods

This study was conducted at the Department of Respiratory Medicine, King George's Medical University, Lucknow, India, after obtaining ethical clearance from the institute's Institutional Ethics Committee (approval number: 124th ECMIIB-Ph.D/P4). The study enrolled both children and adult patients with asthma from the outpatient departments (OPD) of Pediatrics and Respiratory Medicine between 2023 and 2025.

The first group included 96 children aged 6-12 years who visited the OPD of the Department of Pediatrics. They were presenting with symptoms indicative of newly diagnosed asthma and were non-smokers, capable of performing spirometry.

The second group included 136 adolescents and adults aged 13-65 years diagnosed with asthma, from the OPD of the Department of Respiratory Medicine.

Inclusion criteria

Clinically stable patients with no respiratory infection in the past four weeks were included in the study.

Exclusion criteria

Patients with other significant chronic respiratory disorders such as chronic obstructive pulmonary disease (COPD), any cardiac illness, active tuberculosis, congenital lung disease, or any type of malignancy were excluded from the study. Written informed consent was obtained before participation.

Asthma diagnosis was based on a detailed history, clinical examination, and spirometry, which demonstrated an obstructive pattern with reversibility, indicated by an increase in forced expiratory volume in one second (FEV₁). Their socio-demographic data were collected, such as age, gender, height, weight, and body mass index (BMI). Socioeconomic status was assessed through the revised Kuppuswamy scale [15]. Family history of asthma, seasonal variation, and co-existing allergic rhinitis or other atopic diseases, if present, were noted. Smoking history in adults and adolescents was also recorded.

Further, patients were divided into well-controlled, partly controlled, and uncontrolled asthma groups on the basis of the GINA Asthma Symptom Control Test as per GINA 2023 guidelines [16]. This included four questions: (i) frequency of daytime symptoms, (ii) nighttime awakenings, (iii) SABA reliever use for symptom relief, and (iv) any physical activity limitation due to asthma. 

Statistical data analysis

Data of study participants were entered in MS Excel (Microsoft Corp., Redmond, WA, USA), and statistical analysis was done using GraphPad Prism version 8 (GraphPad Software, Inc., San Diego, CA, USA). Categorical variables, such as age, gender, residence, socioeconomic status, asthma control level, atopy history, family asthma history, food allergy, and clinical aspects (e.g., chest tightness, wheezing, dry cough, and exposure to triggers), were expressed using numerical values and percentages.

Mean ± standard deviation (SD) was used to express continuous variables such as age, height, weight, BMI, and spirometric data (FEV₁% predicted, forced vital capacity (FVC), FEV₁/FVC ratio). The Mann-Whitney U test for non-normally distributed data was used to determine differences between the adult and pediatric groups.

## Results

A total of 96 children and 136 adults and adolescents diagnosed with bronchial asthma were enrolled in our study. Among them, the mean age of the study participants was 37.4 years and 8.9 years in the adult and pediatric groups, respectively. Male participants predominated in both groups (adults: 80 (58.8%) vs children: 64 (66.7%)). These socio-demographic characteristics of adults and pediatric asthma patients are shown in Table 1. Urban residence was also common (adults: 100 (73.5%) vs children: 67 (69.8%)), indicating a higher prevalence of asthma in urban areas. Based on socioeconomic status, the lower-middle-income group was most affected by asthma as predicted by the Kuppuswamy socioeconomic scale 2021, as shown in Table 2 [15].

**Table 1:** Socio-demographic characteristics of adult and pediatric asthmatic subjects n: no. of asthma patients

Socio-demographic variables	Adult asthmatic subjects (n = 136)	Pediatric asthmatic subjects (n = 96)
Age in years (mean ± SD)	37.4 ± 12.7	8.9 ± 2.2
Gender, n (%)		
Male	80 (58.8)	64 (66.7)
Female	56 (41.2)	32 (33.3)
Anthropometric measures (mean ± SD)		
Height	158.8 ± 6.8	127.6 ± 14.1
Weight	61.4 ± 7.0	28.1 ± 8.8
BMI	24.2 ± 0.8	17.4 ± 5.2
Marital status, n (%)		
Married	90 (66.2)	NA
Un-married	46 (33.8)	NA
Residence, n (%)		
Urban	100 (73.5)	67 (69.8)
Rural	36 (26.5)	29 (30.2)

**Table 2:** Distribution of patients according to socioeconomic status n: no. of asthma patients

Socioeconomic status	Adult asthma (n = 136), n (%)	Pediatric asthma (n = 96), n (%)
Upper	14 (10.3)	10 (10.4)
Upper middle	24 (17.6)	24 (25)
Lower middle	49 (36)	42 (43.8)
Upper lower	37 (27.2)	14 (14.6)
Lower	12 (8.8)	6 (6.2)

Assessment of clinical characteristics in both asthmatic groups and trigger factors is summarized in Table 3. Nearly half of the patients reported dry cough and wheezing as the most common symptoms found in both adult and pediatric asthma patients. Allergic rhinitis was the most prevalent comorbidity in both groups (adults: 56 (41.2%) vs children: 62 (64.6%)), with the most common occurrence in children, and the prevalence of other comorbidities is also seen as shown in Table 3. Nearly half of the children with asthma (43 (44.8%)) had a family history of asthma (parents/siblings/grandparents) and were atopic (51 (53.1%)). While fewer adults reported a family history of asthma (22 (16.2%)), atopy was observed among half of the adults (67 (49.3%)). These findings indicate that family history was less common among adult patients as compared to children. Adults with asthma showed a lower prevalence of allergic comorbidities compared to children, including allergic rhinitis (41.2% vs 64.6%), eczema (5.1% vs 21.9%), and food allergy (14% vs 25%), which suggests age-related differences in the clinical presentation of the disease between the two groups. Among these patients, we found that recurrent respiratory infections were more common in children than adults (adults: 41 (30.1%) vs children: 53 (55.2%)). In asthma, exacerbations are an important predictor of its severity. Of the 136 adult asthmatics, the majority (106 (77.9%)) had a history of 1-2 times of exacerbation history, whereas 30 (22.1%) had three or four exacerbations, as shown in Table 3. Active smoking was present in 20 (14.7%) adults. Second-hand smoke exposure was reported in eight (8.3%) children and 29 (21.3%) adults.

**Table 3:** Clinical characteristics and triggers of asthmatic adults and children n: no. of asthma patients

Clinical features	Adult asthmatic subjects (n = 136), n (%)	Pediatric asthmatic subjects (n = 96), n (%)
History of atopy	67 (49.3)	51 (53.1)
Family history of asthma	22 (16.2)	43 (44.8)
Age of onset		
Early onset	41 (30.1)	NA
Late onset	95 (69.9)	NA
Food allergy	18 (13.2)	29 (30.2)
Chest tightness	68 (50)	33 (34.4)
Wheezing	76 (55.8)	62 (64.6)
Dry cough	83 (61)	45 (46.9)
Exposure to trigger factors		
Respiratory tract infections	41 (30.1)	53 (55.2)
Pet exposure	24 (17.6)	24 (25)
Dust	31 (22.8)	31 (32.3)
Dampness in the house	34 (25)	13 (13.5)
Pollen exposure	30 (22.1)	12 (12.5)
Smoking		
Active smoking	20 (14.7)	NA
Second-hand exposure	29 (21.3)	8 (8.3)
Biomass exposure	41 (30.1)	10 (10.4)
Frequency of exacerbations		
1-2 per year	106 (77.9)	60 (62.5)
3-4 per year	30 (22.1)	36 (37.5)
Comorbidities		
Allergic rhinitis	56 (41.2)	62 (64.6)
Eczema/atopic dermatitis	7 (5.1)	21 (21.9)
Food allergy	19 (14)	24 (25)
Obesity	18 (13.2)	9 (9.4)

Asthma symptom control test was used to assess the severity of symptoms experienced by the patients according to GINA guidelines which is shown in Table 4 [16]. Out of 136 adult asthma patients, 42 (30.9%) were well-controlled, compared to 26 (27.1%) of 96 pediatric patients (see Table 4). Thus, adults tend to have better control than children. Many patients had partially controlled symptoms, with 70 (51.5%) adults and 43 (44.8%) children, a higher proportion in adults. In Table 5, spirometry tests are depicted which indicate considerably lower FEV₁% predicted and pre-bronchodilator FEV₁/FVC ratios in adults compared with children.

**Table 4:** GINA assessment of asthma control n: no. of asthma patients; GINA: Global Initiative for Asthma

Level of asthma control	Adult asthma patients (n = 136)	Pediatric asthma patients (n = 96)
Well-controlled, n (%)	42 (30.9)	26 (27.1)
Partly controlled, n (%)	70 (51.5)	43 (44.8)
Uncontrolled, n (%)	24 (17.6)	27 (28.1)

**Table 5:** Spirometric assessment of adult and pediatric asthma patients Values are presented as mean ± SD. *Statistically significant (p < 0.05). P-values were compared using the Mann-Whitney U test. FEV₁: forced expiratory volume in one second; FVC: forced vital capacity

Spirometric variables	Adult asthma	Pediatric asthma	P-value (significance)
FEV₁% predicted	63.9 ± 19.4	85.1 ± 16.1	p < 0.0001*
% change in FEV₁	24.6 ± 8.9	16.1 ± 10.7	p < 0.0001*
Pre-FEV₁/FVC	62.6 ± 9.2	79.1 ± 12.8	p < 0.0001*

## Discussion

The present study highlights age-related variations between adults and children with asthma in terms of clinical presentation, socio-demographic profile, asthma control, and lung function. Studies simultaneously analyzing pediatric- and adult-onset asthma remain limited since most available work has investigated these groups individually, emphasizing the importance of a lifespan approach to asthma [17,18]. Allergic asthma usually develops in childhood, while non-allergic asthma worsens with age, consistent with population-based observations by Pakkasela et al. [19]. The association of childhood-onset asthma with allergy and atopy is well-established, as also highlighted by Akar-Ghibril et al. and Foong et al., and is further supported by our findings [20,21].

Pediatric asthmatics in the present study demonstrated a stronger incidence of atopy, including a higher prevalence of family history of asthma, food allergy, allergic rhinitis, and eczema, showing a strong hereditary and atopic susceptibility. In contrast, adult patients more frequently experienced late-onset asthma and more exposure to smoking and biomass fuels, corresponding with the adult-onset asthma phenotype described by de Nijs et al. [22]. Among symptoms, coughing and wheezing were prevalent in all groups. Also, a study by Nandasena et al. found that respiratory tract infections were the most common symptoms in children [23]. Assessment of asthma control using the GINA framework found that most patients in both age groups had partially managed or uncontrolled asthma, a finding similar to global reports from the GINA. Uncontrolled asthma was more common among asthmatic children, which may indicate a higher prevalence of allergy and increased exposure to environmental triggers, as supported by Akar-Ghibril et al. [20]. In contrast, adults generally reported partly controlled asthma, which has been related to the period of the illness and associated comorbidities [22,24].

The prevalence of comorbidities, particularly allergic rhinitis, was common in both adult and pediatric asthma individuals and is known as a key factor leading to poor asthma outcomes [25]. Further, spirometric examination indicated considerably lower FEV₁% predicted and pre-bronchodilator FEV₁/FVC ratios in adults compared with children, reflecting more severe baseline airflow limitation. In research conducted by de Nijs et al. and Ilmarinen et al., airway remodeling and chronic inflammation in long-term asthma were also associated with a similar decline in lung function in adults [22,26]. Conversely, pediatric patients demonstrated significantly retained lung function and increased reversibility, corresponding with findings reported by Trivedi and Denton [17]. Despite having lower baseline lung function, adults showed a stronger bronchodilator response, supporting the findings of Ilmarinen et al. [26] that airway hyperresponsiveness persists even in chronic adult asthma. In summary, our findings reflect how asthma varies among age groups and indicate the need for age-specific assessment and tailored treatment plans.

The key strengths of our study include the enrollment of both pediatric and adult asthma patients, the use of validated and standardized GINA diagnostic criteria, and the incorporation of clinical and environmental assessments. However, the study also has a few limitations. In our study, the cross-sectional design limits the causal relationships. This hospital-based study may lead to selection bias and applicability to the general population. Some self-reported information like past exposures and symptom history can also introduce recall bias in this study.

## Conclusions

This study demonstrates age-related differences in the clinical profile, risk factors, and lung function of asthma patients in a North Indian tertiary care setting. Pediatric asthma was predominantly combined with allergic rhinitis with family history and food allergy suggesting a strong hereditary. In contrast, adults more frequently developed late-onset asthma and non-allergic symptoms and, on spirometry, showed more severe baseline airflow limitation. Asthma control remained suboptimal in both age groups, with the majority of patients having partially controlled or uncontrolled symptoms despite being under specialist care. Uncontrolled asthma was relatively more common in children, whereas adults, though more often partly controlled, demonstrated significantly lower FEV₁% predicted and FEV₁/FVC ratios, reflecting possible airway remodeling and long-standing disease.

Our outcomes highlight the need for age-specific, phenotype-based approaches to diagnosis in asthma management. Control of modifiable environmental risk factors in adults and children and integrated management of comorbidities such as allergic rhinitis are crucial to improving outcomes. Future long-term and biomarker-focused studies from different Indian populations are further needed to refine phenotypic characterization and guide personalized asthma therapy.
